# Current practice of trabeculectomy in a cohort of experienced glaucoma surgeons in Australia and New Zealand

**DOI:** 10.1038/s41433-022-02034-1

**Published:** 2022-05-03

**Authors:** Graham A. Lee, Lance Liu, Robert J. Casson, Helen V. Danesh-Meyer, Peter Shah, ANZGS Trabeculectomy Consensus Group, Ashish Agar, Nina Ashraff, Michelle Baker, Kerr Brogan, Anne Brooks, Alex Buller, Robert Casson, Helen Chan, Jason Cheng, Helen Danesh-Meyer, Guy D’ Mellow, Hamish Dunn, Jennifer Fan Gaskin, Jesse Gale, Ivan Goldberg, Catherine Green, Emily Gregory-Roberts, Paul Healey, Ian Hurley, Tanya Karaconji, Shweta Kaushik, Nathan Kerr, George Kong, Jo Koppens, John Landers, Mitchell Lawlor, John Leaney, Graham Lee, Ridia Lim, Lance Liu, Jed Lusthaus, David Manning, Keith Martin, Katherine Masselos, Nathan Nielsen, Soo Ng, Jon Ng, Divya Perumal, Raj Shah, Kiran Sindhu, Simon Skalicky, Bernardo Soares, Narelle Spencer, Richard J. Symes, Mark Walland, Bob Wang, Andrew White, Joshua Yuen, Sophia Zagora

**Affiliations:** 1 City Eye Centre, Brisbane, QLD Australia; 2 University of Queensland, Brisbane, QLD Australia; 3 Glaucoma Unit, Royal Victorian Eye and Ear Hospital, Melbourne, VIC Australia; 4 Department of Ophthalmology, University of Adelaide, Adelaide, SA Australia; 5 Department of Ophthalmology, Faculty of Medical and Health Sciences, University of Auckland, Auckland, New Zealand; 6 University Hospitals Birmingham NHS Foundation Trust, Birmingham, UK; 7 Department of Ophthalmology, Prince of Wales Hospital, Randwick, NSW Australia; 8 Thorndon Eye Clinic, Lower Hutt, New Zealand; 9 Eyemedics, Wayville, SA Australia; 10 Department of Ophthalmology, Royal Victorian Eye and Ear Hospital, Melbourne, VIC Australia; 11 The Eye Surgery, Hastings, Hawkes Bay New Zealand; 12 Department of Ophthalmology, Royal Adelaide Hospital, Adelaide, SA Australia; 13 Eye Surgery Associates, Melbourne, VIC Australia; 14 Vision Eye Institute, Hurstville, NSW Australia; 15 Department of Ophthalmology, Faculty of Medical and Health Sciences, University of Auckland, Auckland, New Zealand; 16 Terrace Eye Centre, Spring Hill, QLD Australia; 17 Port Macquarie Eye Centre, Port Macquarie, NSW Australia; 18 Capital Eye Specialists, Wellington, New Zealand; 19 Discipline of Ophthalmology, Save Sight Institute, The University of Sydney, Sydney, NSW Australia; 20 Eye Specialist Practice, Bondi Junction, NSW Australia; 21 Centre for Vision Research, Westmead Institute for Medical Research, University of Sydney, Sydney, NSW Australia; 22 Ringwood Eye Specialists, Ringwood, VIC Australia; 23 Centre for Eye Research Australia, Royal Victorian Eye and Ear Hospital, East Melbourne, VIC Australia; 24 Milford Eye Clinic, Auckland, New Zealand; 25 Department of Ophthalmology, Flinders Medical Centre, Adelaide, SA Australia; 26 City Eye Centre, Brisbane, QLD Australia; 27 Hunter Cataract & Eye Centre, Charlestown, NSW Australia; 28 Centre for Eye Health, University of New South Wales, Kensington, NSW Australia; 29 Precision Eye Clinic, Sandy Bay, TAS Australia; 30 Kingswood Eye Centre, Glen Osmond, SA Australia; 31 Eye & Vision Epidemiology Research (EVER) Group, Perth, WA Australia; 32 Eye Institute, Remuera, Auckland, New Zealand; 33 Eastwood Eye Surgery, Eastwood, NSW Australia; 34 Nexus Eye Care, Blacktown, NSW Australia; 35 Mornington Peninsula Eye Clinic, Mornington, VIC Australia; 36 Heidelberg Eye Clinic, Rosanna, NSW Australia; 37 Centre for Vision Research & Westmead Clinical School, University of Sydney, Sydney, NSW Australia; 38 Applecross Eye Clinic, Ardross, WA Australia

**Keywords:** Health care, Health services

## Abstract

**Background/Objectives:**

To evaluate current routine trabeculectomy technique preferences among Australian and New Zealand Glaucoma Society surgeons regularly performing trabeculectomy surgery.

**Subjects/Methods:**

Survey of experienced surgeons who perform trabeculectomy.

**Results:**

Forty-nine surgeons (33 male:16 female) participated in the survey. Trabeculectomy was performed as day surgery (39/47, 83.0%) under local anesthesia (44/47, 93.6%). The surgical techniques most commonly used were a corneal traction suture (44/47, 93.6%), fornix-based conjunctival flap (43/47, 91.5%) and half-thickness scleral flap (38/47, 81.0%). Mitomycin C antifibrotic agent was used in routine cases by 45/46 (97.8%) surgeons. Surgeons applied the antifibrotic agent under the Tenon layer with a pledget (36/46, 78.2%) with a concentration of 0.02% (37/46, 80.4%) for 2 (11/46, 23.9%) or 3 min (30/46, 65.2%). The Kelly (26/46, 56.5%) and the Khaw Descemet (19/46, 41.3%) punches were used to perform the sclerostomy. Most surgeons performed a peripheral iridectomy in all phakic patients (46/47, 97.9%), but less commonly in pseudophakic patients (34/47, 72.3%). Techniques for closure of the limbal conjunctival edge were quite varied with a combination of suturing including purse string (21/47, 57.4%), wing (20/47, 42.6%) and horizontal mattress sutures (33/47, 70.2%). Surgeons reviewed their routine patients four times in the first month (29/47, 61.7%) and continued the postoperative topical steroids for 3–4 months (28/47, 59.6%).

**Conclusions:**

Although a wide range of techniques for trabeculectomy exists among surgeons, there are consistent procedures currently in use to optimize patient outcomes. This report will assist surgeons in choosing which surgical techniques fit their best practice.

## Introduction

Trabeculectomy is designed to divert aqueous from the anterior chamber via a sclerostomy under a scleral flap to an external bleb. Filtration surgery is an option in patients who are using maximally tolerated medications and/or have had laser treatment and require further lowering of their intraocular pressures. It may also be considered as initial management for those presenting with advanced disease [1]. The technique has significantly evolved since it was introduced in 1968 by Cairns [2]. There are a number of different trabeculectomy techniques utilized by ophthalmologists in Australia and New Zealand, largely influenced by their glaucoma fellowship training. Each technique has been modified by individual surgeons according to their experience, preferences and specific clinical features and outcomes of the patients undergoing surgery. While there are numerous surveys that provide preferred practice patterns for glaucoma surgery [3–5], there is a paucity of data regarding preferred surgical techniques for trabeculectomy [6, 7]. The aim of this study is to document the various techniques used by an experienced group of trabeculectomy surgeons in Australia and New Zealand.

## Methods

A survey of glaucoma surgeons attending an online meeting of the Australian and New Zealand Glaucoma Society (ANZGS) (as listed at the end of the paper) was conducted in February 2021. Participation was voluntary and no compensation was offered or provided for responding. The 39-question online survey, divided into five polls, was formulated and modified by the authors after trialing it on four trabeculectomy surgeons.

Participants were asked to recall their technique for a routine primary trabeculectomy performed in an adult. Questions on each step of the trabeculectomy were presented and respondents independently answered via an online polling software (Zoom, San Jose, USA). At the live presentation, after respondents answered each question, the results were displayed and discussed. During the presentation, participants were also able to type in questions and comments in a “chat box”. This commentary was also recorded as part of the survey.

### Data analysis

Data were collected and analyzed anonymously. Respondents reported their technique of choice for each surgical step. These data were collated for all respondents. The total number of responses and percentages are presented. Data from the returned questionnaires were tabulated and analyzed using Microsoft Excel (Microsoft Corporation, Seattle, WA, USA).

## Results

Forty-nine surgeons (33 male:16 female) participated in the study. Most surgeons were aged 30–49 years old (32/49, 65.3%) with the rest, 50–59 years old (14/49, 28.6%) and >60 years old (3/49, 6.1%). The majority did glaucoma fellowships in the UK (43/49, 87.8%) with the rest in the US (3/49, 6.1%) or elsewhere (3/49, 6.1%). The number of years in specialist practice was five or more for 32/49 (65.3%) of the surgeons, with the average number of trabeculectomies performed per year greater than 10 by 36 (73.5%) surgeons and greater than 20 by 21 (42.9%) surgeons. All participants answered the majority of the questions; however, there was a loss of two participants from Polls 2, 4 and 5 and a loss of three participants from Poll 3.

### Surgical technique

The majority of patients prior to trabeculectomy did not have any pre-treatment; however, in 22/47 (46.8%), pre-treatment included topical steroid 16/47 (34.0%) and/or topical pilocarpine therapy 8/47 (17.0%) (Table 1). The vast majority used day surgery for the procedures 39/47 (83.0%). Some surgeons 8/47 (17.0%) used a combination of day surgery or overnight stay; however, only two surgeons used overnight stay most of the time. Monitored local anesthesia with an anesthetist present was used by 44/47 (93.6%) with only two surgeons routinely using general anesthesia 2/47 (4.3%).

**Table 1:** Surgical technique.

Question	*n* (%)
*1. Do you pre-treat your patients prior to trabeculectomy?*	
No	25 (53.2)
Yes	22 (46.8)
*2. Are your patients predominantly day surgery or overnight stay?*	
Day surgery	39 (83.0)
Overnight	0
Combination	8 (17.0)
*3. What anesthetic do you prefer for trabeculectomy?*	
Topical	1 (2.1)
Monitored local	44 (93.6)
General	2 (4.3)
*4. Do you use a traction suture?*	
Yes	44 (93.6)
No	3 (6.4)
*5. Is your conjunctival flap fornix or limbal based?*	
Fornix	43 (91.5)
Limbal	4 (8.5)
*6. What do you use to cut your scleral flap?*	
Diamond knife/crescent blade	14 (29.8)
11-blade/crescent blade	18 (38.3)
Crescent blade only	7 (14.9)
11-blade only	2 (4.3)
Other	6 (12.8)
*7. What is the size of your scleral flap?*	
3 × 3 mm	2 (4.3)
3 × 4 mm	21 (44.7)
4 × 4 mm	8 (17.0)
3 × 4 × 5 mm	4 (8.5)
Other	12 (25.5)
*8. How thick is your scleral flap (thickness of sclera at limbus 800* *µm)?*	
1/4 thickness (approx. 200 µm)	8 (17.0)
1/3 thickness (approx. 260 µm)	38 (80.9)
Other	1 (2.1)
*9. What shape is your scleral flap?*	
Rectangular	32 (69.6)
Square	4 (8.7)
Trapezoidal	9 (19.6)
Triangular	0
Other	1 (2.2)
*10. Do you use anti-fibrotics for trabeculectomy?*	
Mitomycin C	45 (97.8)
5-Fluorouracil	0
Either	1 (2.2)
*11. How do you apply the Mitomycin C/5-Fluorouracil?*	
Soaked pledget under Tenon’s	36 (78.3)
Injection under Tenon’s	8 (17.4)
Onlay over the conjunctiva	0
Subconjunctival injection before opening conjunctiva	1 (2.2)
Subconjunctival injection after closing conjunctiva	0
Other	1 (2.2)
*12. What is your standard Mitomycin C concentration for trabeculectomy with healthy conjunctiva?*	
0.02%	37 (80.4)
0.03%	1 (2.2)
0.04%	7 (15.2)
0.05%	0
Other	1 (2.2)
*13. What is your standard time for Mitomycin C in a trabeculectomy with healthy conjunctiva?*	
1 min	0
2 min	11 (23.9)
3 min	30 (65.2)
4 min	0
N/A	5 (10.9)
*14. Do you place the Mitomycin C under the scleral flap?*	
Yes	17 (37.0)
No	29 (63.0)
*15. How do you suture your scleral flap?*	
Releasable nylon	19 (41.3)
Interrupted nylon	5 (10.9)
Adjustable nylon	6 (13.0)
Combination releasable/interrupted nylon	15 (32.6)
Other	1 (2.2)
*16. When do you suture the scleral flap?*	
Pre-place before the sclerostomy	29 (63.0)
After the sclerostomy has been made	17 (37.0)
*17. How many scleral flap sutures do you use?*	
2	13 (28.3)
3	25 (54.4)
4	6 (13.0)
>4	2 (4.4)
*18. How do you suture your scleral flap?*	
Releasable nylon	19 (41.3)
Interrupted nylon	5 (10.9)
Adjustable nylon	6 (13.0)
Combination releasable/interrupted nylon	15 (32.6)
Other	1 (2.2)
*19. When do you suture the scleral flap?*	
Pre-place before the sclerostomy	29 (63.0)
After the sclerostomy has been made	17 (37.0)
*20. How many scleral flap sutures do you use?*	
2	13 (28.3)
3	25 (54.4)
4	6 (13.0)
>4	2 (4.4)
*21. What do you use to make the sclerostomy?*	
Kelly punch	26 (56.5)
Khaw Descemet punch	19 (41.3)
Handcut	0
Other	1 (2.2)
*22. Do you do a peripheral iridectomy in phakic patients?*	
All cases	46 (97.9)
Most cases	1 (2.1)
*23. Do you do a peripheral iridectomy in pseudophakic patients?*	
All cases	34 (72.3)
Most cases	11 (23.4)
Some cases	2 (4.3)
*24. What is the preferred size of your iridectomy?*	
Small (<1 mm)	14 (29.8)
Medium (1–2 mm)	31 (66.0)
Large (>2 mm)	0
Not important	2 (4.3)
*25. Do you test for flow after scleral flap closure?*	
Yes	45 (95.7)
No	2 (4.3)
*26. What suture do you use to close the conjunctival flap?*	
Monofilament Vicryl	2 (4.3)
Braided Vicryl	4 (8.5)
Nylon	36 (76.6)
Combination	5 (10.6)
*27. What suture technique do you use to close the conjunctival flap (multiple choice)?*	
Purse string	21 (44.7)
Wing	20 (42.6)
Horizontal mattress	33 (70.2)
Interrupted	7 (14.9)
Continuous	3 (6.4)
Other	4 (8.5)
*28. Do you perform a Seidel test at the end of the case?*	
Yes	31 (66.0)
No	16 (34.0)
*29. Do you use subconjunctival antibiotics?*	
Yes	37 (78.7)
No	10 (21.3)
*30. Do you use subconjunctival steroids?*	
Yes	45 (95.7)
No	2 (4.3)
*31. Do you use topical atropine at the end of the trabeculectomy?*	
Yes	19 (40.4)
No	16 (34.0)
Sometimes	12 (25.5)

In terms of surgical technique, most used a corneal traction suture 44/47 (93.6%). Only three surgeons routinely used an Ong glaucoma lid speculum (Epsilon USA, Ontario, California, USA). The majority utilized a superior conjunctival flap that was fornix-based 43/47 (91.5%). There were various techniques to fashion the scleral flap including use of a diamond knife and crescent blade 14/47 (29.8%), 11-blade and crescent blade 18/47 (38.3%), crescent blade only 7/47 (14.9%) and 11-blade only 2/47 (4.3%). The scleral flaps ranged in size, with the most common size being 3 × 4 mm 21/47 (44.7%) and half-thickness of the sclera (400 µm) 38/47 (80.9%). The flap was either rectangular 32/46 (69.6%), trapezoidal 9/46 1(9.6%) or square 4/46 (8.7%). The majority of surgeons used mitomycin C (MMC) 45/46 (97.8%), applied as a pledget under the Tenon layer 36/46 (78.3%) at a concentration of 0.02%—37/46 (80.4%). There were a number of surgeons who utilized injection of the MMC under the Tenon capsule 8/46 (17.4%). The most common exposure times for MMC were 3 min 30/46 (65.2%) or 2 min 11/46 (23.9%). Most 29/46 (63.0%) surgeons did not place MMC under the scleral flap. The scleral flap sutures were pre-placed before the sclerostomy in 29/46 (63.0%), with 25/46 (54.4%) using three stitches and 13/46 (28.3%) using two stitches.

The most common instrument for making the sclerostomy was a Kelly punch 26/46 (56.5%); however, the Khaw Descemet punch 19/46 (41.3%) was also popular. There were no surgeons who handcut the sclerostomy. The peripheral iridectomy was performed by surgeons in phakic patients very commonly 46/47 (97.9%), however, less commonly in pseudophakic patients at 34/47 (72.3%) in all cases and 11/47 (23.4%) in most cases. The size of the peripheral iridectomy was usually around 1–2 mm—31/47 (66.0%) or smaller at less than 1 mm—14/47 (29.8%). A common technique was to test the flow after scleral flap closure 45/47 (95.7%). The most common type of sutures to close the conjunctiva were 10/0 nylon 36/47 (76.6%) with other stitches used including either monofilament or braided 9/0 polyglactin 910 6/47 (12.8%) or a combination 5/47 (10.6%). The suturing technique for the conjunctival flap was quite variable with surgeons using purse-string sutures 21/47 (44.7%), wing sutures 20/47 (42.6%) and/or horizontal mattress 33/47 (70.2%). The majority of surgeons performed a Seidel test at the end of conjunctival closure 31/47 (66.0%). In terms of intraoperative medications, a subconjunctival antibiotic was used in 37/47 (78.7%), subconjunctival steroid in 45/47 (95.7%) and topical atropine in 19/47 (40.4%).

Most surgeons do not posture the patient on day one post-op 43/47 (91.5%). Patients were reviewed either three or four times in the first month 43/47 (91.5%). Topical steroids were mostly used in the first three to six months 40/47 (85.1%). Postoperative subconjunctival 5-fluorouracil anti-scarring injections were routinely performed in 17/47 (36.2%). There was no use of adjunctive devices such as the ExPRESS (Alcon Laboratories Inc, Fortworth, Texas, USA) or Ologen implant (Aeon Astron Europe BV, Leiden, The Netherlands).

## Discussion

Although the practice patterns for trabeculectomy have been studied by a number of surveys [6–8], these studies did not address current practice preferences regarding surgical techniques for trabeculectomy in detail. This study aimed to evaluate current practice techniques for trabeculectomy among Australian and New Zealand glaucoma specialists. While the results of this survey demonstrate a wide range of techniques in trabeculectomy among glaucoma surgeons, it also suggests that there are many consistent features in surgical techniques that are undertaken by the majority of surgeons. This group of surgeons practice mainly in private clinics but have some public hospital appointments and/or academic positions. According to Australian Medicare statistics, there were 1987 trabeculectomies performed in the 12 months from July 2019 to June 2020 [9].

Pre-treatment with topical steroids was a routine part of trabeculectomy for a third of surgeons 16/47 (34.0%). It was used particularly in eyes with ocular surface inflammation and a history of previous uveitis. Most trabeculectomies were performed under monitored local anesthesia 44/47 (93.6%) as a day procedure 39/47 (83.0%), indicating the trend away from general anesthesia in routine adult patients that has occurred progressively over the past 20 years [10]. Trabeculectomy requires attention to detail at every step to optimize results and minimize postoperative complications. Areas of the surgical technique where surgeons had overwhelming consistency (>90%) included use of a corneal traction suture 44/47 (93.6%), fornix-based conjunctival flap 43/47 (91.5%), application of MMC 45/46 (97.8%), peripheral iridectomy in phakic patients 46/47 (97.9%), testing the flow after scleral flap closure 45/47 (95.7%), intraoperative injection of subconjunctival steroid in 45/47 (95.7%) and review three to four times in the first month postoperatively 43/47 (91.5%).

A corneal traction suture is an effective way of infraducting the eye to provide good exposure of the superior bulbar conjunctiva. Placing a superior rectus suture, although previously one of the most techniques [10], often results in subconjunctival hemorrhage or superior rectus hematoma that may trigger an excessive wound healing response in trabeculectomy [11]. The Ong eyelid speculum has a larger inferior blade that is able to push on the inferior conjunctival fornix resulting in the downwards rotation of the eyeball. This is effective in most eyes, although surgeons may still need a traction suture in some cases.

The Moorfields Safer Surgery System for Trabeculectomy was formulated by Professor Sir Peng Khaw [11]. This has had a direct influence on ANZGS surgeons’ technique as many of the surgeons in this survey were trained in the United Kingdom either under Peng Khaw himself or by one of his many fellows. The key features of this system are to form a diffuse posteriorly draining bleb with normal conjunctival morphology. The use of MMC-soaked pledgets placed over a wide area under the Tenon layer, pre-placed scleral flap sutures, a small sclerostomy, intraoperative IOP titration and watertight closure of the limbal incision all contribute to the success of this technique [11]. The ability to control the fibrosis and allow long-term drainage of the bleb has revolutionized trabeculectomy surgery. The concentration of MMC depends on the anticipated scarring response of the conjunctiva and Tenon’s layer, with a greater risk of fibrosis in younger patients, Asian/African-Caribbean ethnicity, previous surgery or active intra- or extraocular inflammation. Generally, a higher dose of MMC such as 0.03–0.04% for 3–4 min is indicated in cases at high risk of scarring and filtration failure. In a recent study by Seol et al., a concentration of 0.02% compared to 0.04% for 2 min resulted in no difference in efficacy or safety in a South-East Asian population; however, follow-up was for only 6 months [12]. The widespread use of antimetabolites in this survey is in contrast to the National Survey of Trabeculectomy in the United Kingdom conducted in 1996, where no antimetabolite was used in 83.6% [10].

The scleral flap has evolved over many years with various techniques [13]. The flap shape can be square, rectangular or trapezoidal and is generally a third to half thickness of the sclera. The purpose is to regulate the fluid egress from the anterior chamber to allow adequate pressure control, but also to prevent hypotony. Pre-placed scleral flap sutures allow for a rapid closure of the scleral flap reducing the intraoperative time of hypotony. A thicker flap (half thickness of the sclera) was preferable to reduce flap complications. Placement of the MMC under the flap also reduces closure from fibrosis. The application of the MMC varied from sub-Tenons pledgets (preferably polyvinyl alcohol [14]), injection at the beginning of the case and injection following dissection of the conjunctival flap. Injection of MMC is a less controlled technique than direct sponge application. Intravitreal and subconjunctival bevacizumab may also be used as an adjunctive agent to reduce postoperative fibrosis [15].

There are several areas where there is significant variability. The use of an anterior chamber maintainer was quite popular amongst the more recently trained of the group. The infusion of fluid maintains the anterior chamber after the sclerostomy so the eye pressure can be adjusted by titrating the flow through the scleral flap [11]. The size of the scleral flap was quite variable, with the other popular choice measuring 5 × 3 mm. Another one of the most variable steps in trabeculectomy technique was the closure of the scleral flap. There was a combination of fixed, releasable and/or adjustable sutures used. Fixed sutures often needed to undergo laser suture lysis postoperatively, whereas adjustment or removal of releasable sutures can be performed at the slit lamp [11]. Adjustable sutures utilizing a triple, quadruple or quintuple throw can be loosened postoperatively to titrate the flow rather than an “all or nothing” effect with releasable sutures. However, manipulation can be difficult through an edematous and inflamed conjunctiva and there is a risk of buttonholing the conjunctiva. Closure of the conjunctiva and Tenon’s layer is critical to prevent wound leaks and postoperative hypotony. The variability in technique indicates there are many ways to achieve this result. The main principle is to avoid postoperative bleb leaks, especially following the use of an antifibrotic agent.

Postoperative guttae atropine 1% 19/47 (40.4%) was in common use and can relax the ciliary muscle with pain relief, may reduce postoperative bleeding, can reduce anterior chamber shallowing as well as reducing the risk of aqueous misdirection, stabilizes the blood aqueous barrier and prevents central posterior synechiae. Disadvantages of its use include a dilated pupil, which may blur vision and increase lens-corneal touch in shallow anterior chambers [11].

There are several limitations of this survey. A multiple-choice format may introduce bias by limiting possible responses to the options offered. However, the choices were carefully designed to include the most common techniques for each step and the chat box discussion further elucidated other options. Furthermore, answers given by some respondents may not accurately reflect their actual clinical practice because of errors in recall. Given that the results were anonymously collected, this would be unlikely. The study included most of the higher volume trabeculectomy surgeons in Australia and New Zealand, although it was not possible to obtain the opinions of every experienced trabeculectomy surgeon in the region. Ideally, trabeculectomy outcomes of all the surgeons involved could be compared to the various techniques in order to determine the most efficacious techniques. There was no attempt to compare the technique to trabeculectomy outcomes; however, the techniques used have been well documented with efficacy established [10].

The significant variations in practice preferences of trabeculectomy techniques among Australian and New Zealand glaucoma surgeons reflect a lack of good evidence to guide practice or maybe it reflects what works clinically, given the variable outcomes that one sees in our glaucoma surgery patients. Although the optimal clinical practice is poorly defined, the results of this survey provide a means for ophthalmologists to critically examine their own clinical preferences through comparison with those of their colleagues and adopt different techniques, given the variable nature of each eye’s response to glaucoma surgery. This survey also provides a baseline against which clinical trends can be identified and assessed in future. It may stimulate consideration of a registry-base and potential randomized controlled trials that could lead to refinement of surgical techniques to improve success rates, ultimately reducing the complications of this highly effective surgical procedure.

### Summary

#### What was known before

- A wide range of techniques for trabeculectomy exists amongst surgeons.
- There are consistent procedures currently in use to optimize patient outcomes.

#### What this study adds

- Areas of the surgical technique where surgeons had overwhelming consistency included use of a corneal traction suture, fornix-based conjunctival flap, application of mitomycin C, peripheral iridectomy in phakic patients, intraoperative injection of subconjunctival steroid and review three to four times in the first month postoperatively.

## Data Availability

Data are available upon reasonable request from the authors
